# Death scene investigation and autopsy proceedings in identifying the victims of the terror attack on the Breitscheidplatz in Berlin 19^th^ December 2016

**DOI:** 10.1007/s12024-020-00277-6

**Published:** 2020-07-31

**Authors:** Claas Buschmann, Sven Hartwig, Michael Tsokos, Lars Oesterhelweg

**Affiliations:** Institute of Legal Medicine and Forensic Sciences, Charité - Universitätsmedizin Berlin, Berlin Institute of Health, Freie Universität Berlin, Humboldt-Universität zu Berlin, Turmstr. 21, Haus N, 10559 Berlin, Germany

**Keywords:** Forensic autopsy, Disaster victim identification (DVI), Death scene investigation, Blunt trauma, Casper’s sign

## Abstract

We describe and discuss the forensic mission after the terrorist attack on the Breitscheidplatz in Berlin on 19th December 2016, focusing on co-operation with police authorities, and the injury patterns of the deceased. Even after massive blunt trauma, severe injury patterns are often unrecognizable by visual inspection of the body (“Casper’s sign”), which could instill false security among rescuers or, as happened on the Breitscheidplatz, may lead to distress or even trauma in rescue personnel when obviously primarily uninjured patients die suddenly.

## Introduction

**Fig. 1:**
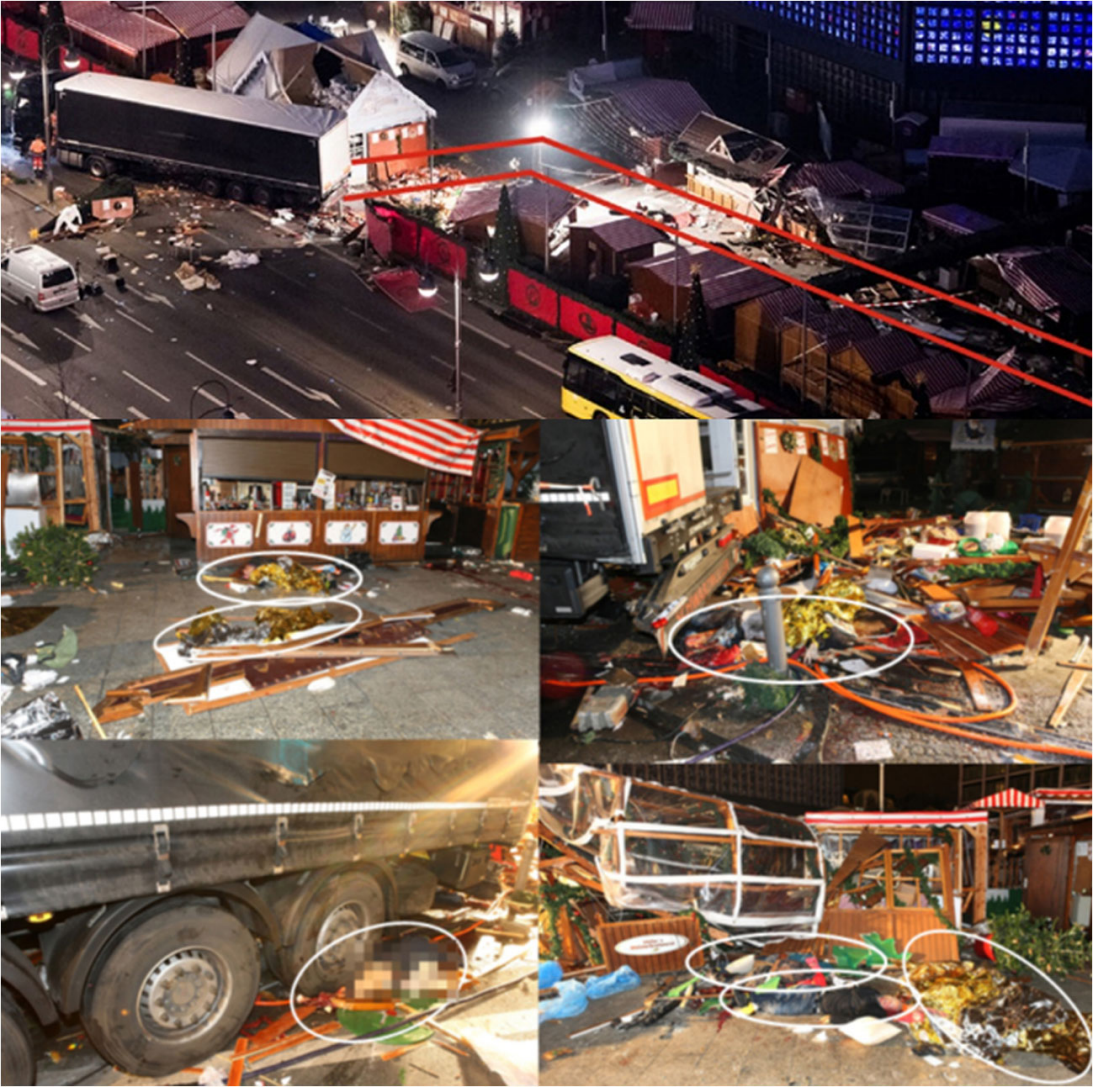
“Corridor” of the truck (from [1]) with overview of the positions of the deceased in the “corridor” and under the truck

On 19th December 2016 at 08:02 pm, 24-year-old Anis Amri drove a previously hijacked 40-ton semitrailer into a crowded Christmas market at the Breitscheidplatz in Berlin, Germany, at a speed of approximately 60 – 70 km/h (Fig. 1). A short video sequence of the incident is available at https://www.youtube.com/watch?v=GipGqdvZy0o. Anis Amri had hijacked the truck prior to this action by shooting its driver (the so-called “co-driver” as he was found on the passenger seat of his truck, see below).

## Crime scene investigation

On 19th December 2016 at approximately 08:45 pm, the forensic on-call service was contacted by the Co-ordination Centre of the Berlin State Office of Criminal Investigation to alert them to the incedent. In addition to the two regular-duty colleagues (foreground and background), a request was made for “several colleagues” to proceed to the location of the incident. Initially, the police raised the alarm with the forensic “foreground” service; further alarms proceeded by private telephone chain. At approximately 09:30 pm, five forensic colleagues (three assistant doctors and two forensic specialists) arrived at the scene. At this time (approximately 90 min after the attack), the pre-hospital treatment of the injured market visitors was complete, and no injured persons were present at the scene. It was recognizably a police situation instead of an emergency medical situation. The pre- and in-hospital aspects of the management strategies for the medical treatment of the injured persons have been reported elsewhere [1, 2].

After establishing a police management structure for the particular scenario, crime scene work by the police and forensic pathologists began at approximately 1:00 am. First, extensive forensic evidence collection and securement were performed. After an initial inspection of the scene, seven deceased were found freely accessible behind the truck in a “corridor” along which it had literally “cut” through the market, and two more bodies were trapped under the truck (under the tractor and under the semi-trailer, respectively, which was loaded with 25 tons of steel). Eight of the deceased bodies were complete, one had been partly dismembered by blunt force. Three more severely injured victims died the same night in various hospitals in Berlin despite cardiopulmonary resuscitation attempts and emergency surgery.

First, the entire scene (corridor and truck with semitrailer) was divided into five areas. Five teams were formed, each consisting of a photographer from the Berlin State Office of Criminal Investigation, a forensic pathologist, an evidence securer, and an investigator from the homicide squad.

The first priority was the forensic examination of the body of the “co-driver”, who had been brought out of the truck immediately after the incident and had undergone futile resuscitation attempts. Rescue personnel reported to the police that the co-driver had a head injury, which had to be investigated and classified. The head injury was confirmed as a gunshot wound during the crime scene work later that night. Once it was confirmed that the co-driver had been shot in the head, it was obvious that the incident was a terrorist attack (instead of a traffic accident, which had been considered as a possiblity by the police until then).

Second, the remaining eight bodies were recovered and some preliminary attempts at identification were initiated. This proceeded at the site of the incident until the early morning hours of 20th December 2016.

Except for the “co-driver”, all eight deceased presented with severe blunt torso and extremity trauma at post-mortem examination at the scene, some with extensive décollement injuries (“Morel-Lavallée lesion”) [3]. Only one deceased had an isolated open traumatic brain injury pattern, which occurred after rollover by the truck. Apart from this deceased, the other dead victims were at least provisionally identified *prima vista* using personal documents.

With the exception of the deceased with the open traumatic brain injury from the truck rollover trauma and the two deceased trapped under the truck (initially inaccessible), extensive cardiopulmonary resuscitation attempts had been performed on the victims, with attempts at bleeding control using tourniquets in cases of décollement injuries to the legs.

## Autopsy findings

**Fig. 2:**
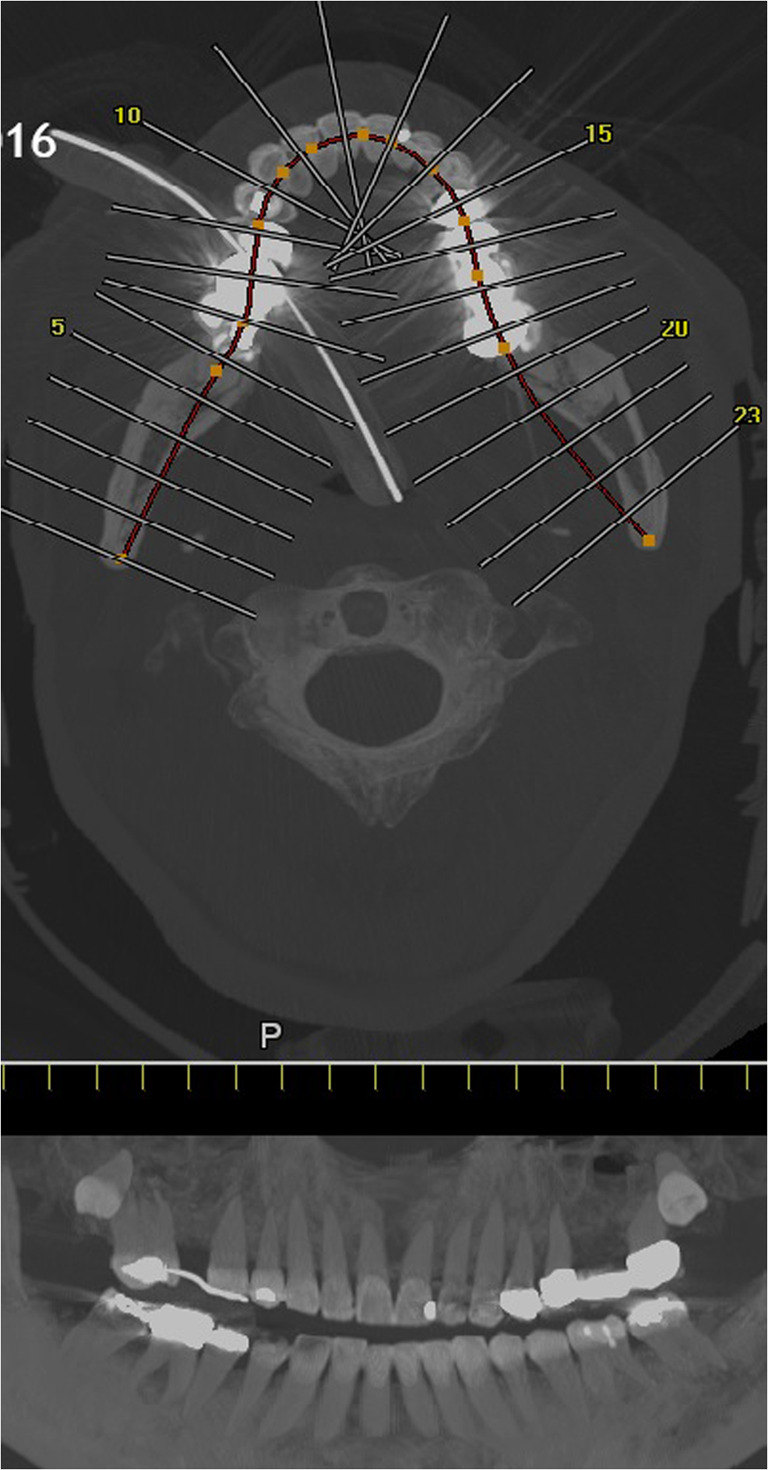
Dental reconstruction for identification purposes according to post-mortem computed tomography data

**Fig. 3:**
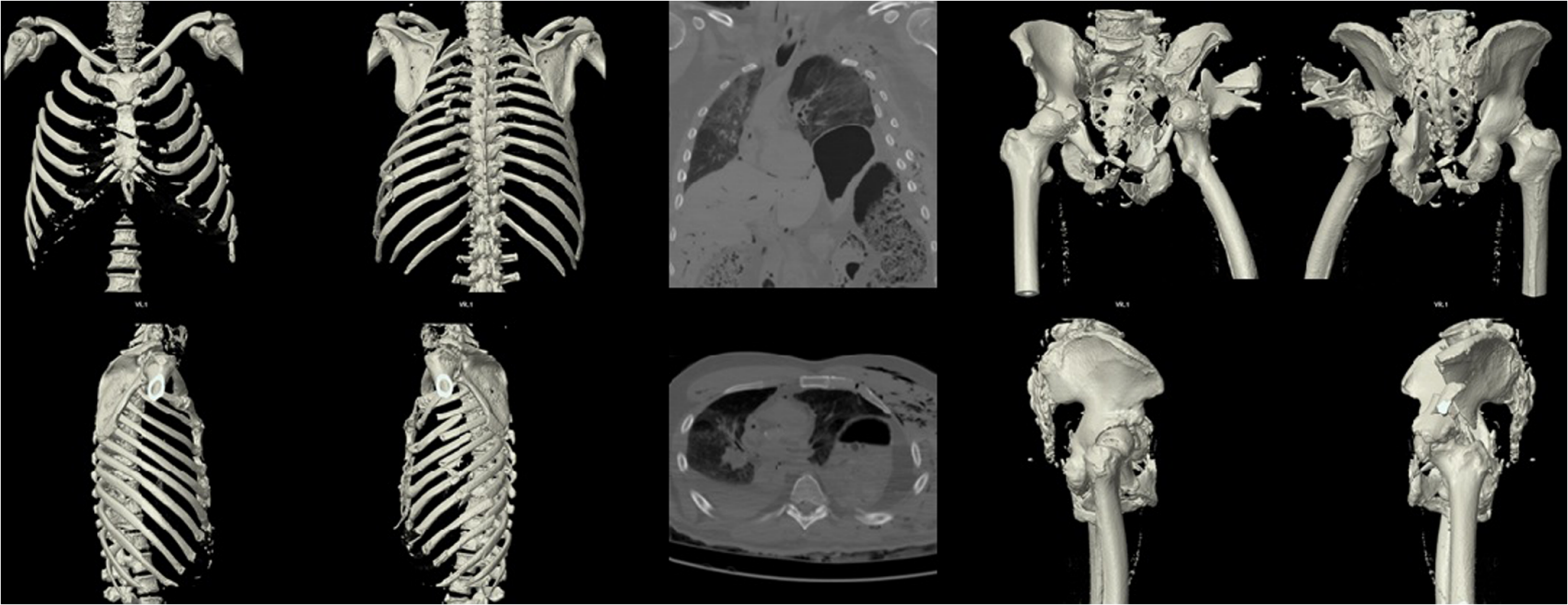
Example of the injury patterns of the deceased obtained from the post-mortem computed tomography data showing severe chest and pelvic trauma after truck rollover/impact trauma

**Fig. 4:**
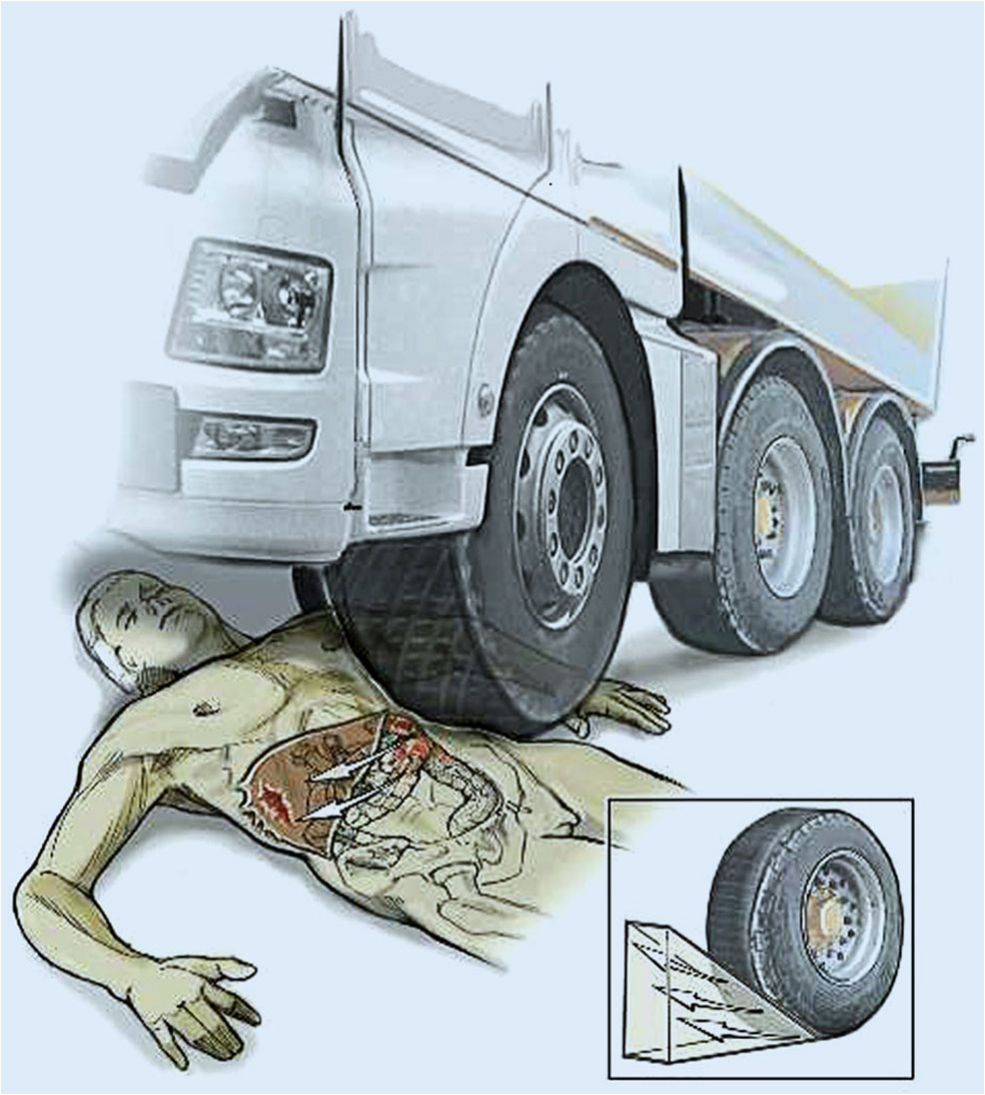
Representative example of an injury pattern after truck rollover showing incomplete rollover trauma with massive left-sided crush injuries to the trunk. On the contralateral side, the displacement of the internal organs caused the right liver lobe to burst (from [1])

On the morning of 20th December 2016, an autopsy was performed on the “co-driver”, confirming the fatal gunshot injury to the head. From afternoon of 20th December 2016 to late evening of 22nd December 2016, under the auspices of the Identification Commission of the Federal Criminal Police Office, the autopsies of the remaining 11 deceased (including the three patients who died in-hospital) were performed according to International Standards for the Identification of Victims of Mass Disasters (Disaster Victim Identification [4]). This included data obtained by post-mortem computed tomography; for example, for dental reconstruction (Fig. 2). Characteristic injury patterns of the market visitors were consistent with massive blunt trauma (as commonly seen in traffic accidents in routine forensics) (Fig. 3). Combined severe blunt thoracic, abdominal, and pelvic trauma patterns were present, some with gross destruction of the extremities and the presence of Morel-Lavallée lesions (Table 1). It should be noted that the predominant external appearance of the deceased exposed to massive blunt trauma by truck rollover (apart from the décollement injuries) was that they were relatively uninjured, while at autopsy, massive destruction of the internal organs was noted in accordance with the injury mechanism (Fig. 4).

**Table 1:**
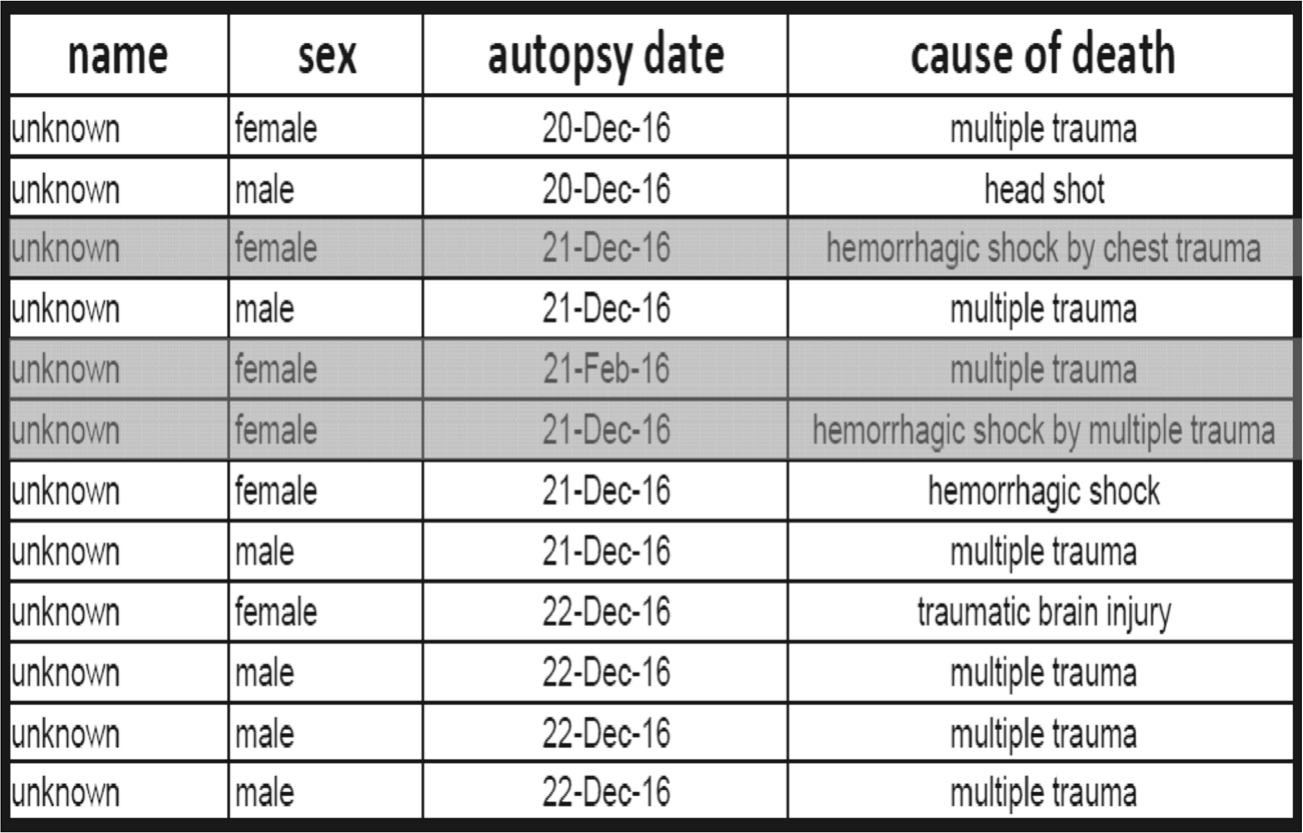
Overview of the causes of death after autopsy. The three patients highlighted in gray died in-hospital the night of the attack; the remaining nine patients died at the scene

## Discussion

Terror attacks conducted using vehicles, especially trucks with high weight and heavy additional load, are not uncommon [1]. Typically in such vehicle-ramming attacks four different injury mechanisms occur:

1. *Direct impact trauma from collision with the vehicle:* Through direct contact with the vehicle, the victim’s body sustains numerous fractures and often severe craniocerebral injuries. Dependent on the speed at the time of impact, deceleration injuries of internal organs may also occur.
2. *Throwing away:* The victim collides with the vehicle and is thrown away. The extent of the throwaway depends on the mass and the speed of the vehicle, and on the density of the affected crowd. Impact with the ground can also cause limb fractures and severe traumatic brain injury.
3. *Roll-over trauma:* Because of the high vehicle weight, massive crush injuries occur, accompanied by tissue tears, i.e. decollement injury, and complex comminuted fractures.
4. *Secondary trauma through evasive maneuvers and compression within the crowd:* When trying to evade the approaching vehicle, victims can sustain secondary injuries which can also occur outside the immediate vicinity movement. In addition, bystanders can be injured or compressed by fleeing people – similar to a mass panic – and by obstacles being pushed away. The injury pattern in these cases is highly variable.

Autopsies in our cases revealed direct injury patterns resulting from mechanisms 1–3, but no secondary trauma. The finding that, especially after blunt trauma, severe internal injury patterns are often not immediately recognizable *prima vista* without further physical examination is known as *“Casper’s sign”* (first described by Johann Ludwig Casper, 1796–1864, forensic physician in Berlin) [5, 6]. Nonetheless, apart from the fatal gunshot wound to the head seen in the “co-driver”, the 11 other victims, including the three that dies in the hospital, initially presented with non-survivable multiple trauma patterns after massive blunt trauma caused by truck rollover/impact. Potentially or definitively preventable trauma deaths from omitted emergency measures in the pre- or in-hospital setting [7, 8] were not found. Likewise, none of the deceased had hypothermia-related findings at autopsy (0 °C outside temperature at the time of the attack) secondary to delayed rescue or delayed transport to hospital. All deaths occurred immediately or shortly after the attack, and no further deaths of initially surviving injured market visitors occurred. As is standard in capital offenses, forensic examination of the surviving victims of the attack were not ordered by the Federal Prosecutor’s Office.

In February 2017, seven weeks after the incident, the Berlin Fire Department held an interdisciplinary, non-public symposium for rescue personnel and police responding to the incident entitled “Lessons Learned”. In addition to the discussion of emergency medical and police strategies regarding the attack scenario, autopsy findings were presented to the audience, which provided both forensic information regarding injury mechanisms and patterns, but also considerable psychological relief; emergency medical treatment had been fast and sufficient. The experience gained from the terrorist attacks in Boston, Paris, Madrid and Mumbai have led to concepts for pre-hospital mass casualties (not only in case of terrorist attack) that were proved to work in reality, at least from a forensic point of view. Despite the rarity of experiences with terrorist attacks in Germany, co-operation between the fire department, police authorities, and forensic medicine was excellent.

Performing the autopsies in the Institute of Legal Medicine and Forensic Sciences of the Charité – Universitätsmedizin Berlin benefitted from the fact that the autopsies of 10 German tourists killed in a bomb attack in Istanbul/Turkey in January 2016 were performed 10 months before in a similar personnel constellation [9].

## Permission of the Federal Prosecutor’s Office

We obtained permission from the Federal Prosecutor’s Office to publish the autopsy findings in anonymous and blinded form for scientific purposes.

## Key points

1. The risk of terrorist attacks in Germany has become a reality.
2. Forensic autopsy in mass disaster victims should include regular use of post-mortem computed tomography for identification purposes (i.e. dental work, implants) prior to dissection.
3. Severe blunt, i.e. fatal trauma is not always obvious at postmortem examination (“Casper’s sign”).
4. Forensic autopsy diagnosis can provide psychological relief for rescue personnel in cases of sufficient emergency medical treatment in traumatic deaths, e.g. terrorist attacks.
